# Hydrodynamic Cavitation-Based Pretreatment of Primary Digestate: Feasibility and Role in Overall Enhancement of Valorization

**DOI:** 10.1021/acsomega.5c04230

**Published:** 2025-08-06

**Authors:** Jagdeep Kumar Nayak, Vivek V. Ranade

**Affiliations:** Department of Chemical Science, 8808 Bernal Institute, University of Limerick, Limerick V94 T9PX, Ireland

## Abstract

Hydrodynamic cavitation (HC) is a promising biomass pretreatment technology for enhancing methane production from anaerobic digestion (AD). While HC has been widely studied for sludge and fresh biomass feedstock, its application to digestate remains largely unexplored. This study evaluates the impact of HC pretreatment on digestate from different sources to enhance biochemical methane potential (BMP) and biodegradability. The results demonstrate a significant increase in methane yield, with the BMP increase ranging from 100 to 200% after HC pretreatment. The biodegradability index improved almost 3 times, and sCOD increased >20% for all of the digestate samples, indicating enhanced substrate accessibility. Energy analysis revealed that the methane production energy yield (*E*_Yield_) exceeded the HC-based pretreatment energy input (*E*_HC_), with the highest *E*_Yield_/*E*_HC_ ratio of 9.18 at 40 passes, confirming HC as an energy-efficient strategy. The BMP data was interpreted by using appropriate kinetic models, which included the lag phase. The results demonstrated for the first time that HC pretreatment reduces lag phase and improves the rate as well as the BMP of primary digestate. This study provides valuable insights into the potential of HC pretreatment for digestate pretreatment to enhance biomethane recovery from the secondary digester and highlights its feasibility as an energy-efficient solution for improving the overall performance of AD plants employing secondary digesters.

## Graphical abstract

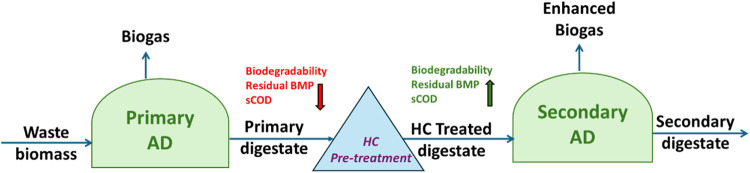

## 1 Introduction

There is a growing interest in the circular economy approach, particularly in fostering industrial synergies that integrate multiple technologies to improve both energy generation and waste management.^1^ Among several technologies, anaerobic digestion (AD) processes capture methane emissions from decomposing organic matter, preventing greenhouse gases from entering the atmosphere. This mitigates climate change impacts and contributes to a more sustainable environmental economy. The biogas produced can be utilized for electricity, for heat, or as a vehicle fuel, reducing reliance on fossil fuels. Additionally, the resulting digestate is a valuable fertilizer, enhancing soil health and reducing the need for chemical fertilizers.^2^

There are two primary types of AD systems, i.e., dry and wet. Dry AD operates with higher total solids content, typically above 20%, while wet AD functions with lower solids content, usually below 15%. Although dry AD can reduce the volume of digestate produced, it presents several challenges. One significant issue with dry AD is the accumulation of ammonia and volatile fatty acids (VFAs) due to the high total solids content. This accumulation can inhibit microbial activity, leading to reduced biogas production and process instability.^3^ The reduced water content in dry AD systems can also cause gas and liquid diffusion problems, further hindering microbial processes.^4^ Operational challenges, such as difficulties in leachate recirculation and unstable biogas production during biomass addition and removal, have also been reported in dry AD systems. Moreover, insufficient retention time in dry AD can result in incomplete digestion, decreasing biogas yields and potentially destabilizing the digester.^5^ In rural settings, wet AD systems are often preferred due to their simpler design and ease of operation. Small-scale wet AD digesters can be operated as batch systems, making them more suitable for rural applications.^6^ Furthermore, wet AD systems have lower operation and maintenance requirements compared to multistage systems, making them more practical for rural communities.^7^ The application of digested slurry from wet AD as fertilizer can significantly reduce odors and flies in rural areas, improving local air quality and public health.^8^

While AD offers numerous environmental and economic benefits, managing the resulting digestate presents several challenges that require careful consideration. According to the report prepared by the World Biogas Association in 2021, around 132,000 small, medium, and large-scale anaerobic digesters currently operate worldwide, with approximately 50 million microscale digesters serving households and small communities.^9^ The improper management of digestate can lead to environmental concerns, including eutrophication, harmful algal blooms, and hypoxia in water bodies, ultimately reducing oxygen levels and threatening aquatic ecosystems.^10^ Recent advancements in AD valorization through digestate management have introduced innovative strategies to enhance both economic and environmental outcomes. Various approaches have been implemented to address residual methane generation from digestate, including solid–liquid separation, digestate recirculation, and a range of pretreatment methods: physical, chemical, biological, and combined. These strategies are designed to improve the breakdown of complex organic materials, thereby boosting biogas yields, shortening retention times, and improving overall process efficiency.^11,12^ However, many of these pretreatment methods have not been widely adopted due to their high-energy requirements, the need for specialized equipment, and the generation of numerous byproducts as a result. Therefore, mechanical pretreatment methods, such as hydrodynamic cavitation (HC), could offer a more energy-efficient alternative, as demonstrated by previous studies.^13,14^

HC has emerged as an effective pretreatment technique to enhance the digestibility of biomass and digestate, significantly improving biomethane yields in the AD processes. HC disrupts complex organic structures by generating localized high-energy conditions through bubble formation, expansion, and collapse, increasing the substrate bioavailability for microbial action. For instance, HC pretreatment of wheat straw using a rotor-stator assembly significantly increased methane yields from 31.8 to 77.9 mL/gCOD_VFA_ L^–1^. When combined with potassium hydroxide (KOH) treatment, the biogas yields further increased to 172.3 mL/gCOD_VFA_ L^–1^. Additionally, HC pretreatment of urban waste, including thickened sewage sludge and vegetable waste, resulted in an 83% increase in soluble chemical oxygen demand (sCOD) and elevated volatile fatty acids (VFAs) concentration from 1.9 to 17.3 gCOD_VFA_ L^–1^, enhancing the subsequent acidogenic fermentation process.^15,16^ Vortex-based devices have gained prominence among various HC technologies due to their energy efficiency and scalability. Unlike conventional cavitation designs such as orifices, venturi, or rotor-stators, vortex-based HC systems initiate cavitation at lower pressure levels and experience significantly less material erosion and pressure drop, leading to reduced overall energy consumption and enhanced operational longevity.^17^

A typical arrangement of two commonly used digesters in series is used in practice for processing a wide range of waste biomass streams (shown schematically in Figure 1a). Overall valorization may be enhanced by using HC pretreatment either to the feed of the primary digester (shown schematically in Figure 1b) or to the primary digestate. When feed streams are hard to digest, like dairy sludge or wastewater sludge, option 1b is preferred.^18^ When the AD feed stream is relatively easy to digest, such as food waste or energy crops like Napier grass, option 1c is preferred. In this work, we focus on option 1c and explore the potential for valorizing primary digestate using HC pretreatment by evaluating its residual biogas production and assessing its economic feasibility. The analyzed results and data are presented in this study. This study presents a novel approach to applying HC directly to primary digestate to enhance residual methane recovery. It demonstrates improvements in biodegradability, methane yield, and net energy gain, establishing HC as a practical and energy-efficient pretreatment method. To the best of the author’s knowledge, such a detailed evaluation of HC for primary digestate valorization has not been reported in the open literature. HC-based pretreatment is identified as a promising strategy to enhance digestate valorization and economic feasibility within the biogas plant ecosystem.

**Figure 1:**
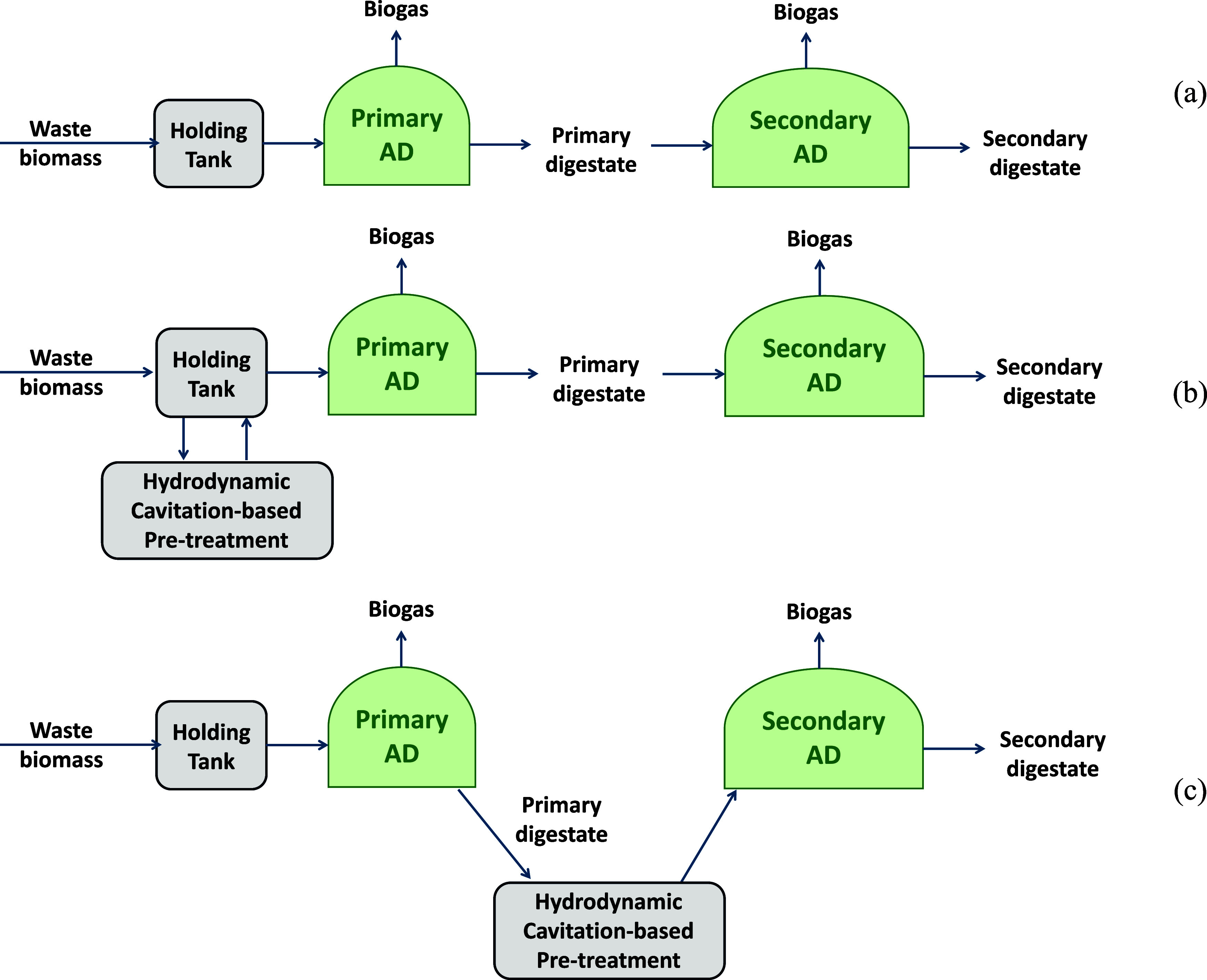
Possible induction of HC pretreatment technology in the AD ecosystem, (a) typical two digesters in series, (b) HC-based pretreated feedstock for biogas enhancement, and (c) HC-based pretreatment of digestate to maximize residual methane production.

## 2 Materials and Methods

### 2.1 Digestate Collection and Characterization

The three primary digestate samples were collected from AD plants in Limerick, Ireland. The prime feedstocks for digestates 1, 2, and 3 are sludge, grass silage, and food waste, respectively. The nature of the collected digestates is highly dependent on the feedstocks and the operational conditions. The samples were stored at 4 °C to avoid further methanogenesis and methane production. The digestate samples were characterized according to the standard method.^19^ Total COD (tCOD), soluble COD (sCOD), total phosphorus (TP), and total nitrogen (TN) were analyzed using Hach-Lange cuvette tests (LCK014/LCI400, LCK350, and LCK338 kit, respectively) from HACH, Germany. All measurements were performed with a DR1900 spectrophotometer (Hach). The elemental composition (C, H, N, and S) of the samples was determined using a Vario EL cube elemental analyzer, while the oxygen content was calculated based on ISO 16948. The characterization results for the samples are presented in Section 3.

### 2.2 HC Pretreatment Setup and Biochemical Methane Potential Measurements

A bench-scale vortex-based HC pretreatment system with a nominal flow rate of 20 LPM was employed for the pretreatment of the digestates. The additional details of the HC device are the same as those reported by Islam and Ranade.^20^ The experimental setup used in this work is shown schematically in Figure 2.

**Figure 2:**
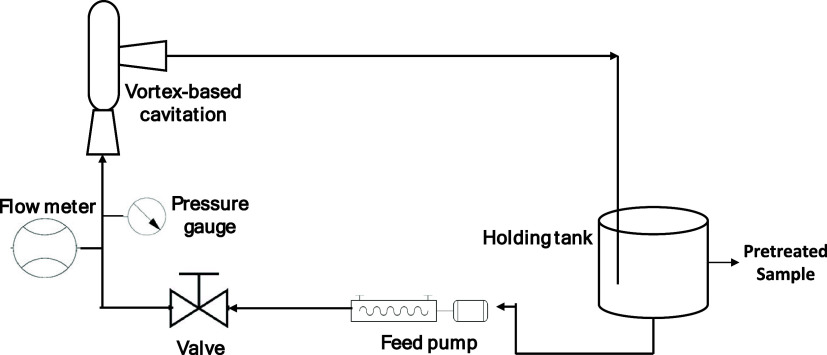
Schematic diagram of HC pretreatment setup.

The setup comprised a progressive cavity pump, flow meter, pressure gauge, and vortex-based HC device. The digestate as received was diluted to prepare samples with 1% volatile solids (VS). Eight liters of such diluted digestate were charged into the holding tank. The pretreatment was carried out by operating the HC device with a pressure drop of 250 kPa. Samples were collected after 20, 40, and 80 passes. The temperature of the digestate samples during HC pretreatment was 20 ± 2 °C. All treated and untreated samples were subsequently characterized and utilized for the BMP analysis. BMP studies were conducted on pretreated and untreated samples using an AMPTS II system from BPC Instruments (Lund, Sweden). The BMP measurements were conducted using 15 reactors, each with a 400 mL reaction capacity, maintained at a constant temperature of 39 °C in a water bath. The inoculum-to-substrate VS ratio was set at 2:1. Mixing was performed intermittently, with the stirrer operating at 90 rpm for 30 min, followed by a 30 min pause in a continuous cycle throughout the experiment. Biogas produced in each reactor was transferred via Tygon tubing into a bottle containing 80 mL of 3 M NaOH to absorb CO_2_ and H_2_S. Additionally, 5 mL of 0.4% thymolphthalein pH indicator was added to a 1 L NaOH solution to monitor the absorption capacity. After the removal of CO_2_ and H_2_S, the purified gas, primarily methane, was directed to the respective flow cell unit for volume measurement, with data continuously monitored on a computer.

All experiments, including the control, were conducted in triplicate. Methane production was determined by subtracting the methane generated by the inoculum from the total methane produced by the inoculum–substrate mixture. Methane yield was expressed in milliliters per gram of volatile solids (mL/g of VS).

### 2.3 Kinetic Models of Residual Methane Production

In this study, cumulative methane production over a 32-day anaerobic digestion process was interpreted and analyzed using various kinetic models. Based on this preliminary analysis, the modified Gompertz (MG) model (eq 1) and the logistic model (eq 2) were found to represent the experimental data very well (as evidenced by their low root mean square error (RMSE) values). Several previous studies have employed similar approaches for interpreting biogas production data.^21–23^ The following equations were used to describe the BMP data^24^

1 $$G=G_{\max}\exp\{-\exp\left[\frac{R_{m}e}{G_{\max}}\left(\lambda -1\right)+1\right]\}$$

2 $$G=\frac{G_{\max}}{1+\exp\left[\frac{{4R}_{m}}{G_{\max}}\left(\lambda -t\right)+2\right]}$$

where *G*_max_ represents the maximum biogas yield over time (mL/g-VS), *G* denotes the ultimate biogas potential (mL/g-VS), *R*_m_ is the maximum daily biogas production rate (mL/g-VS), λ signifies the lag phase duration (days), *e* is the Euler’s constant (2.7183), and *t* refers to the digestion time (days). The interpretation of experimental BMP data and the fitted values of model parameters are discussed in Section 3.2.

## 3 Results and Discussion

### 3.1 Pre- and Post-HC Characterization of Digestates

The physicochemical characteristics of three different digestates (Digestate 1, Digestate 2, and Digestate 3) were analyzed in terms of TS, VS, COD, and TOC (Figure 3a). Figure 3b presents other key parameters, such as TP, TN, ammonium, and orthophosphate. The pH of the digestate samples was between 7.5 and 8.5, indicating a stable, slightly alkaline condition favorable for methanogenesis. Well-stabilized digestates typically exhibit mild alkaline pH, with even higher values observed in the solid-separated fraction. These slightly alkaline pH levels primarily result from the degradation of volatile fatty acids (VFAs) and the production of ammonia (NH_3_) during anaerobic digestion.^25^ Among the digestates, TS ranged from 70 to 100 g/L, while VS varied between 3 and 6% of dry weight. The variation in the TS and VS contents among the digestates indicates differences in feedstock composition and digestion efficiency. Another study suggested the range of TS between 47 and 287 g/kg when using industrial, agri-based waste, and pig and cattle manure.^26^ Similarly, the COD values ranged from 70 to 110 g/L, suggesting differences in the remaining undigested organic matter. The previous studies indicated an even lower COD of 0.2–29 g/L in digestate obtained with organic waste mixture feedstock.^27^ The TOC of all digestate samples ranged between 20 and 30 g/L. The low TOC in all digestates indicates substantial degradation of readily biodegradable organic matter during primary anaerobic digestion. However, TOC alone may not capture recalcitrant yet degradable fractions, suggesting the need for advanced post-treatment strategies like HC or codigestion to enhance residual methane recovery. The BMP_th_ was calculated using the Boyle’s equation using the C, H, N, S, and O values from the analyzer.^18^ The range of BMP_th_ of the three digestates was between 500 and 700 mL/g-VS. Though the theoretical BMP values estimated from elemental analysis look high, most of the remaining value is locked as recalcitrant, difficult-to-digest matter. The ratio of BMP_exp_/BMP_th_ suggested the biodegradability index (further discussed in Section 3.2).

**Figure 3:**
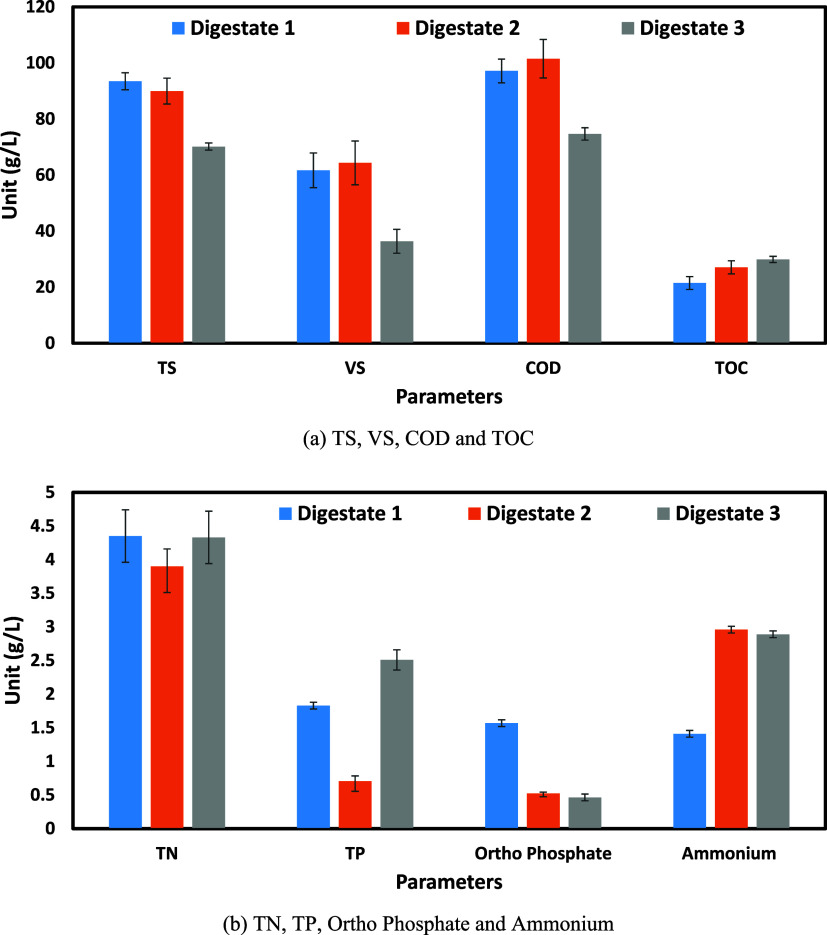
Characterization of the digestates: (a) solid and organic matter characteristics of different digestates. (b) Nutrient composition of digestates of different digestates.

### 3.2 Effect of HC Pretreatment on sCOD

The effect of the HC-based pretreatment on digestate solubilization was evaluated by measuring the soluble COD (sCOD) with different passes across different digestate sources. The results showed a significant increase in sCOD with the number of HC passes (20, 40, and 80), indicating enhanced organic matter solubilization. Among the tested digestates, digestate 3 exhibited the highest sCOD increase of 21% after 80 passes, followed by digestates 2 (13%) and 3 (12.5%). The increase in the level of sCOD suggests that HC effectively disrupts particulate organic matter, improving its bioavailability for subsequent microbial degradation. Most studies have focused on enhancing sCOD and the degree of disintegration in fresh waste biomass.^13,18,28^ A study on sludge disintegration using a rotor-stator-type HC system reported an increase in sCOD from 34 to 633 mg/L, achieving a solubilization rate of 42.3%.^29^ Similarly, research by Habashi and team^30^ showed that HC pretreatment of waste-activated sludge led to increased biogas production, attributing this enhancement to improved organic matter solubilization. However, very few studies have explored digestate disintegration using mechanical- and chemical-based pretreatments, possibly due to concerns over economic viability and the inherent resistance of digestate to further degradation. An increase of 11% sCOD was noticed with ultrasonic-based manure digestate pretreatment, which is comparable to this study.^31^ An increase of nearly 50% in sCOD was observed when chemico-thermal treatment combined with maceration was applied to the solid fraction of cow manure digestate.^32^ Another approach of the combined ultrasonic and ozone pretreatment resulted in changes in the characteristics of the liquid digestate along with sCOD.^33^ These findings are consistent with the observed increase in sCOD following HC-based pretreatment in this study, reinforcing the potential of HC to enhance digestate solubilization and boost biogas production. Furthermore, the nutrient composition of the digestates, including TN, TP, orthophosphate, and ammonium, remained relatively stable, indicating minimal nutrient loss during the HC pretreatment. These findings suggest that HC treatment can enhance digestate utilization in anaerobic digestion by improving organic matter solubilization while preserving essential nutrients. The increase in sCOD with the number of passes through the HC device may be represented as eq 3

3 $${\mathrm{sCOD}}^{*}=\frac{\mathrm{sCOD}}{{\mathrm{sCOD}}_{0}}=e^{\Phi n}$$

where sCOD* is the ratio of sCOD value after *n* passes through the HC device and the initial sCOD (sCOD_0_), and Φ is the per-pass performance parameter of the HC device operated as in this work. It can be seen from Figure 4 that eq 3 represents the experimental data reasonably well.

**Figure 4:**
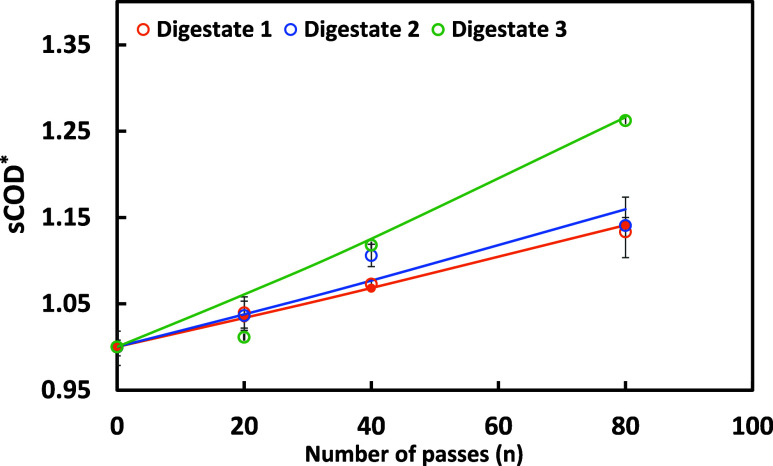
Experimental (symbols) and predicted (solid lines), indicating the effect of HC pretreatment on sCOD* in different passes. The sCOD* is calculated based on eq 3.

The HC device’s effectiveness and per-pass performance of the HC reactor are discussed in more detail by Ranade.^34^ The Φ values ranged from 1.5 × 10^–3^ to 3 × 10^–3^, indicating a notable per-pass impact of HC on sCOD. The increase in sCOD indicates that HC treatment will have a beneficial impact on digestibility and recoverable methane from the treated digestate. This is discussed in the following section.

### 3.3 Effect of HC Pretreatment on Biomethane Production

The influence of HC pretreatment on cumulative methane production (mL/g-VS) over time for different pretreatment intensities (0, 40, and 80 passes) for digestate was analyzed and is presented in Figure 5a–c. The overall BMP enhancement (%) of digestates upon different passes (40 and 80) is shown in Figure 5d. The presented data indicate that HC pretreatment enhances methane production. The extent of increase was found to increase with the number of passes through the HC device. However, there is not much of an increase between the 40 and 80 passes. For Digestate 1, methane production increased by over 195% after 40 passes and further rose to 230% after 80 passes. The biodegradability index improved from 0.063 to 0.21, highlighting the potential of HC pretreatment for effective valorization. Digestate 2 exhibited a 102% increase in methane production, with its biodegradability index improving from 0.09 to 0.18. Similarly, Digestate 3 showed a maximum methane production increase by 97%, with the biodegradability index rising from 0.11 to 0.23. The observed enhancement in BMP of pretreated digestate using a vortex-based HC device was found to be significantly higher than other reported results. For example, thermal pretreatment of digestate was found to improve the biomethane production during postdigestion by 21–22% (food waste-digestate) and 9% (agricultural waste-digestate).^35^ Similarly, ultrasonic pretreatment to agricultural digestate was found to result in a 21% increase in methane production,^36^ while Azman^31^ reported an 8% increase.

**Figure 5:**
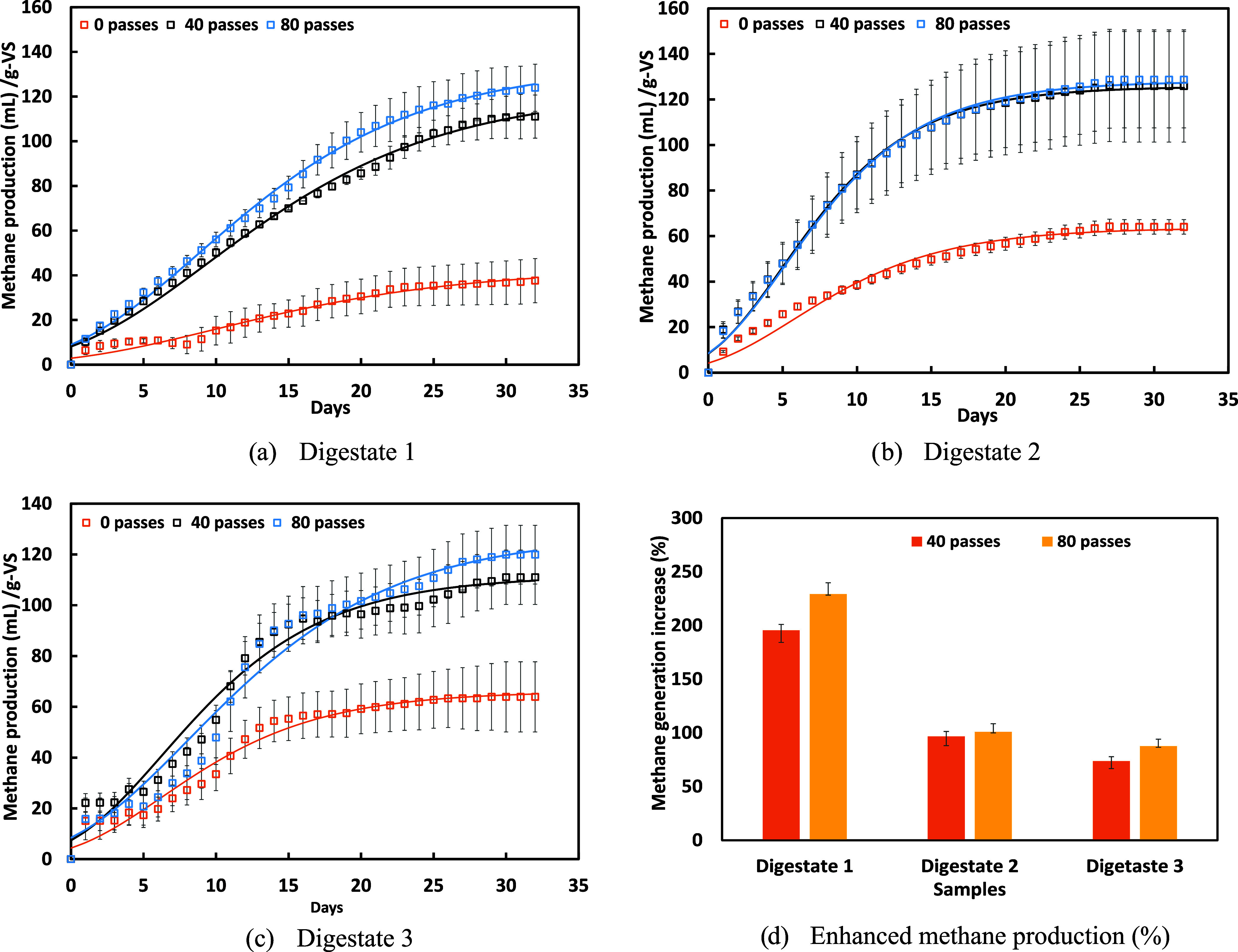
(a–c): Cumulative residual methane production and kinetic models of raw and pretreated HC digestate sample. The continuous lines indicate a fitted MG model with parameters listed in Table 1, and (d) increased methane generation of pretreated digestate with different passes.

The increase in methane production during the initial days for the 80 passes sample suggests enhanced hydrolysis and microbial activity. The 40 passes sample shows a notable improvement compared to the untreated sample, with a further increase observed at 80 passes. Several studies have demonstrated the effectiveness of HC as a pretreatment method to enhance the BMP and AD processes. Earlier studies have revealed that for easier-to-digest biomass streams, a number of passes between one and ten are adequate,^37,38^ while for difficult-to-digest biomass streams, 40 to 80 passes are needed.^18,20^ Considering that the easy-to-digest portion of the feed is already digested in the primary anaerobic digester, the primary digestate is difficult to digest. Therefore, the HC pretreatment with fewer than 40 passes was not investigated. Based on these considerations, in the present work, the effective number of passes was selected as 40 and 80. The current study also showed that increasing the number of passes from 40 to 80 resulted in only an ∼10% improvement in methane yield, while doubling the energy input resulted in a diminishing return at higher intensities. Therefore, experiments beyond 80 passes were not conducted.

A systematic evaluation of MG and logistic models for describing BMP kinetics revealed that the MG model provided the best fit (*R* ^2^ > 0.98) for biomethane generation across all of the considered (with and without pretreatment) digestate samples. The fitted parameters of the MG model are given in Table 1. Three sets of parameters were obtained for zero (without pretreatment), 40, and 80 passes through the HC device. The lag phase duration (λ) was found to be zero for all of the cases. The maximum daily biogas production rate (mL/g-VS), *R*_m_, was found to depend very strongly on the number of passes. The maximum methane production (*G*_max_) was found to increase with the number of passes. The correlation of *G*_max_ with the number of passes has been stated in eq 4. The highest *G*_max_ of 135 mL/g-VS was recorded for 80 passes of the digestate 1 sample. Similarly, digestates 2 and 3 have the maximum predicted methane production of 128 and 122 mL/g-VS recorded, which was close to the experimental value. Regarding the maximum biogas production rate, digestate 1 showed a *R*_m_ of 5.7 mL/g-VS at 80 passes, representing a 3.5-fold (255%) increase. Digestates 2 and 3 exhibited nearly 2-fold (125%) increase in *R*_m_ at 80 passes from 0 passes. A previous study showed an increase of 24.8% *R*_m_ using mechanical pretreatment (ball mill) in the straw digestate, which is lower compared to the HC pretreatment developed in this work.^39^ The enhanced methane production rates observed with HC-pretreated digestate are primarily due to improved microbial accessibility to biodegradable organic matter, such as cellulose and hemicellulose. The variation in the percentage increase is dependent on the properties and biodegradation characteristics of each digestate. However, the observed increasing trend suggests that HC positively impacts the biodegradability and bioavailability of digestate, enhancing residual methane production. The lag phase duration (days) for all of the digestates was zero. The near-zero lag phase indicates the digestate’s readiness for biomethane production. The presence of essential active methanogenic communities enables the immediate generation of methane. Overall, the results indicate that HC pretreatment effectively improves methane yield in all digestates. The magnitude of improvement varies between different digestates, likely due to differences in the substrate and digestate composition. Further analysis is needed to assess the energy balance and economic feasibility of increasing pretreatment intensity for large-scale applications.

**Table 1:** MG Model Kinetic Parameters of Raw and Pretreated Digestate Samples (λ = 0 for All of the Samples)

sample	passes	*R*_m_ (mL/g-VS d^–1^)	*G*_max_ (mL/g-VS)
digestate 1	0	1.6	43
	40	4.8	123
	80	5.7	135
digestate 2	0	4	64
	40	9.2	126
	80	9.1	128
digestate 3	0	3.9	66
	40	6.5	112
	80	7.1	122

Based on this study, the correlation between maximum methane production and the number of passes may be established as (eq 4)­

4 $$G_{n}=G_{\max}-\left(G_{\max}-G_{0}\right)e^{-0.032n}$$

where *G*_*n*_ is the maximum methane production (mL/g-VS) after pretreatment with *n*, the number of passes through the HC device, *G*_max_ is the maximum methane production (mL/g-VS) of the samples, and *G*_0_ is the maximum methane production (mL/g-VS) at zero passes. Equation 4 will be useful for identifying an optimum number of passes through the HC device.

### 3.4 Net Energy Gains via HC Pretreatment

The net energy gain for a specific pretreatment condition is evaluated by accounting for the additional biomethane produced and the energy consumed during the pretreatment process. This study analyzed the energy benefits of HC-based pretreatment for primary digestate. The energy required for HC-based pretreatment (*E*_HC_) is determined using eq 5.

5 $$E_{HC}=\frac{\Delta \mathrm{Pn}}{3600\times \eta \times \rho }\left(kWh/m^{3}\right)$$

where Δ*P* is the pressure drop (250 kPa), n is the number of passes through the cavitation device, η is the pump efficiency (considered as 70%), and ρ is the density of digestate (985 kg/m^3^). Energy gained (*E*_yield_) by HC pretreatment is calculated using eq 6

6 $$E_{yield}=\Delta H_{cal}y_{VS}{\left[\Delta G_{\max}\right]}_{\left(pretreated‐untreated\right)}\;\left(kWh/m^{3}\right)$$

where Δ*H*_cal_ is a typical electricity generation capacity of methane (10 kWh/m^3^), *y*_VS_ indicates the VS of the digestate sample (0.06 ton/ton digestate), and Δ*G*_max_ indicates the cumulative methane generation (mL).

The net energy gains were calculated by comparing the additional energy produced (*E*_Yield_) because of the pretreatment with the energy consumed for the HC pretreatment (*E*_HC_). These reported values of net energy gains may be used for estimating the overall economics by considering local costs. Initially, the BMP values without HC treatment were the lowest, with digestate 1 at 37 m^3^/ton VS, digestate 2 at 64 m^3^/ton VS, and digestate 3 at 63 m^3^/ton VS. After 40 passes, a notable increase was observed, with BMP enhancement reaching 74, 62, and 48 m^3^/ton VS for digestates 1, 2 and 3, respectively (Figure 6). Increasing the number of passes to 80 further improved BMP, but the rate of increase with *n* slowed, indicating a diminishing return with an additional number of passes. Energy effectiveness, measured as the ratio of energy gained (*E*_Yield_) to energy consumed in pretreatment using HC (*E*_HC_), showed that digestate 1 had the highest effectiveness at 40 passes (*E*_Yield_/*E*_HC_ = 9.18), followed by digestate 2 (7.69) and digestate 3 (7.14). However, at 80 passes, the effectiveness decreased for all digestates, with digestate 1 to 5.39, digestate 2 to 4.24, and digestate 3 to 3.97. This suggests that while an additional number of passes continues to enhance BMP, its energy effectiveness becomes less favorable beyond a certain threshold. If the relevant data on capital costs of HC device and other operating costs is available, eq 4 may be used for optimizing the number of passes that can lead to minimum overall cost over the lifecycle of the plant. The presented data and approach will be useful for estimating and enhancing the viability of HC-based pretreatment for industrial-scale applications.

**Figure 6:**
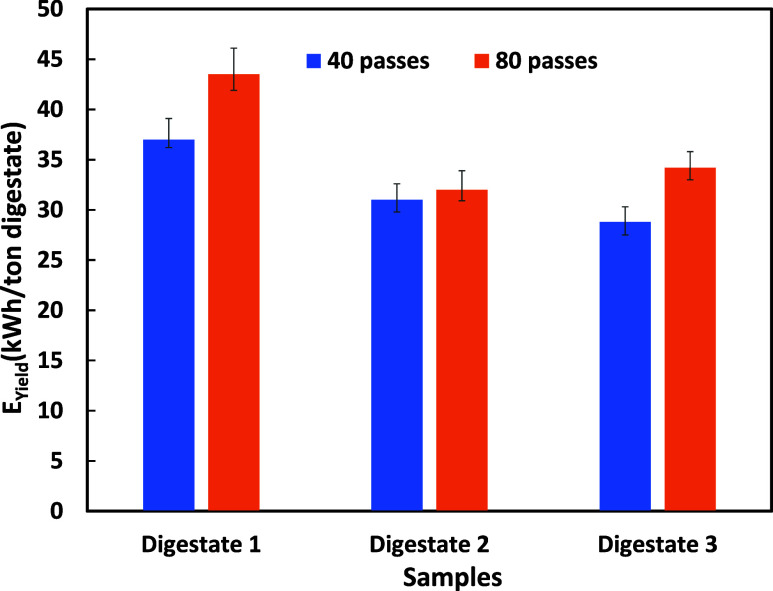
Net energy gain from digestate with HC pretreatment.

HC pretreatment significantly improves BMP, with 40 passes providing the optimal balance between methane production and energy efficiency. Several studies have evaluated the energy benefits of using HC as a pretreatment method to enhance biogas production. A full-scale implementation of HC pretreatment in an agricultural biogas plant resulted in a 10% increase in specific methane production with the input of 0.130 kWh/kgTS.^40^ Another study investigated the application of HC in the disintegration of aerobic granular sludge, focusing on the energy balance associated with different pretreatment durations. The findings indicated that a 15 min HC pretreatment yielded the highest net energy gain of 0.226 Wh/gTS (226 kWh/MgTS). This suggests that optimizing the HC pretreatment process is crucial for maximizing energy benefits in biogas production processes.^41^ The net energy yield from this study indicates that the HC pretreatment could be an energy-efficient pretreatment technology not only for fresh feedstock but also for digestate valorization.

## 4 Conclusions

This study investigated the impact of HC pretreatment on digestate to enhance BMP and overall valorization performance. The results of the developed HC pretreatment demonstrated significant improvements in biodegradability and BMP across different digestate sources. Key conclusions from this work are

- HC pretreatment improved biodegradability and increased sCOD, indicating better substrate availability for microbial degradation.
- HC pretreatment led to a 100–200% increase in residual methane production from the digestate.
- The BMP data were well represented by the modified Gompertz kinetic model.
- The techno-economic analysis suggested a positive net energy gain, with the highest *E*_Yield_/*E*_HC_ ratio of 9.18 observed for digestate 1 at 40 passes.

These findings suggest that HC pretreatment is a promising strategy for digestate valorization.

## Data Availability

The data underlying this study are available in the published article.
